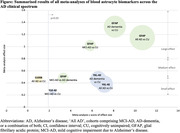# Blood astrocyte biomarkers in AD: a systematic review and meta‐analysis

**DOI:** 10.1002/alz.090544

**Published:** 2025-01-09

**Authors:** Sarah Holper, Paula M Loveland, Leonid Churilov, Dominic Italiano, Rosie Watson, Nawaf Yassi

**Affiliations:** ^1^ The Walter and Eliza Hall Institute of Medical Research, Parkville, VIC Australia; ^2^ The Royal Melbourne Hospital, Parkville, VIC Australia; ^3^ Melbourne Brain Centre at the Royal Melbourne Hospital, The University of Melbourne, Parkville, VIC Australia

## Abstract

**Background:**

We performed a systematic review and meta‐analysis of blood astrocyte biomarkers in Alzheimer's disease (AD).

**Method:**

MEDLINE and Web of Science were searched without any restrictions on language, time, or study design, for studies reporting blood levels of the astrocyte biomarkers GFAP, YKL‐40, and S100B in AD patients. Pooled effect sizes were determined using Hedge's g method with a random effects model. The review was prospectively registered on PROSPERO (registration number CRD42023458305).

**Result:**

The search identified 1186 studies; 36 met inclusion criteria (n=3366 AD patients, n=4115 cognitively unimpaired [CU]). Compared to CU individuals, AD patients had significantly higher GFAP and YKL‐40 levels (GFAP effect size 1.15, 95% CI 0.94‐1.36, P<.0001; YKL‐40 effect size 0.38, 95% CI 0.28‐0.49, P<.0001). Both biomarkers showed a trend towards a greater effect size in more advanced disease. No significant difference in blood S100B levels were identified.

**Conclusion:**

Our findings reveal significant elevations in blood GFAP and YKL‐40 levels in AD patients across the clinical spectrum, suggesting that both biomarkers accurately reflect AD‐related pathology.